# Gastrodin exerts robust neuroprotection in the postischemic brain via its protective effect against Zn^2+^-toxicity and its anti-oxidative effects in astrocytes

**DOI:** 10.1080/19768354.2018.1549099

**Published:** 2018-11-30

**Authors:** Lidan Luo, Seung-Woo Kim, Hye-Kyung Lee, Il-Doo Kim, Hahnbie Lee, Ja-Kyeong Lee

**Affiliations:** a Department of Anatomy, Inha University School of Medicine, Incheon, Republic of Korea; b Medical Research Center, Inha University School of Medicine Incheon, Incheon, Republic of Korea; c Department of Biomedical Sciences, Inha University School of Medicine, Incheon, Republic of Korea

**Keywords:** Gastrodin, MCAO, anti-Zn^2+^-toxicity, astrocyte, neuroprotection

## Abstract

Gastrodin (GAS) is a predominant bioactive constituent of the Chinese herbal medicine Tianma (*Gastrodia elata* Blume). Many authors have reported GAS has the beneficial effect on diverse diseases of the CNS, including epilepsy, Alzheimer’s disease, Parkinson’s disease, and cerebral ischemia. Here, we report GAS exhibited a robust neuroprotective effect in an Sprague-Dawley rat model of stroke (transient middle cerebral artery occlusion, tMCAO), and show that the underlying molecular mechanism involves its protective effect against Zn^2+^-toxicity and its anti-oxidative effects in astrocytes. Intraperitoneal administration of GAS (40 mg/kg) after MCAO reduced mean infarct volume to 30.1 ± 5.9% of that of MCAO controls and this neuroprotective effect was accompanied by neurological function recoveries which was measured using modified neurological severity score (mNSS). Interestingly, GAS induced up-regulation and nuclear translocation of Nrf2, and subsequently increased the expressions of anti-oxidative genes, such as, HO-1 and GCLM, in astrocytes. Furthermore, GAS co- or pre-treatment markedly suppressed Zn^2+^-induced cell death caused by excessive ROS production and PARP-1 induction. We found that GAS suppressed p67 expression and PAR formation in astrocytes, which might underlie the anti- Zn^2+^-toxicity and anti-oxidative effects of GAS in astrocytes. These findings indicate GAS protects astrocytes from Zn^2+^-induced toxicity and oxidative stress and these effects contribute to its neuroprotective effects in the postischemic brain.

## Introduction

Gastrodin (GAS) is the main phenolic compound derived from the roots of Gastrodia elata Blume, which has been used in East Asia to treat epilepsy, paralysis, vertigo, headache, and convulsions (Park et al. 2011). It has been reported that GAS exerted neuroprotective effects in rat model of cerebral ischemic injury (Liu et al. 2016). GAS is able to cross the blood brain barrier (BBB) where it is metabolized to p-hydroxybenzyl alcohol (Lin et al. 2008). In a previous study, GAS inhibited oxygen-glucose deprivation (OGD)-induced calcium and nitric oxide increases in hippocampal neuron (Zeng et al. 2006) and enhanced cell survival under hypoxic conditions by inhibiting excitotoxicity (Xu et al. 2007). In addition, in LPS-stimulated microglial cells, GAS exerted anti-inflammatory effects by inhibiting the phosphorylations of IkB and mitogen-activated protein kinase (MAPK) (Dai et al. 2011). Interestingly, long-term GAS treatment was found to improve memory impairment induced by IDPN (3,3'-Iminodipropionitrile), by normalizing the serotoninergic system (Wang et al. 2015). Various molecular mechanisms have been proposed to explain the anti-oxidative effects of GAS, these include, attenuation of catalase reduction, increased superoxide dismutase (SOD) expression (Zhao et al. 2012) and the translocation of Nrf2 (nuclear factor erythroid-2-related factor) (Wang et al. 2014)

Zn^2+^ acts as a cofactor of various enzymes and transcription factors. In the central nervous system (CNS), Zn^2+^ accumulates in synaptic vesicles in neuron, and this accumulation maintains intracellular Zn^2+^ concentrations via ZnT3 (Cole et al. 1999). However, it has been reported that after transient forebrain ischemia, Zn^2+^ accumulation induces neuronal injury, and that this injury can be suppressed by chelating Zn^2+^ (Koh et al. 1996). Excessive Zn^2+^ release from neurons has a neurotoxic effects because it inhibits glyceraldehyde-3-phosphate dehydrogenase (GAPDH) and thus, reduced glycolysis and NAD production (Sheline et al. 2000). In addition, protein kinase C (PKC)-dependent intracellular ROS elevation has been reported in Zn^2+^-treated cortical neurons (Noh et al. 1999). Zn^2+^-mediated toxicity has also been reported in glial cells, for example, Zn^2+^-mediated damage has been reported in astrocytes, and putatively attributed to ROS production and the inhibition of glutathione reductase (Bishop et al. 2007). In addition, Zn^2+^ released from neurons was observed to induce PARP-1 activation in astrocytes and to subsequently suppress glutamate uptake by astrocytes and aggravate neuronal injury (Suh et al. 2007).

Astrocytes have diverse functions, which include maintenance of fluid and ion balance, axon regeneration, neurotransmitter regulation, and energy substrate, neurotransmitter precursor, and growth factor release. In particular, in the postischemic brain, astrocytes regulate ischemic tolerance (Rossi et al. 2007). For example, astrocytes release glutamate and ATP, which contribute to neuroprotection by acting on sensors of ischemic tolerance in mildly ischemic brain (Li et al. 2008). Astrocytes also express NADPH oxidase, which activated by PKC and intracellular calcium and modulated by intracellular pH and play pivotal roles in pathophysiology of the CNS (Abramov et al. 2005). Interestingly, exacerbation of brain damage in GFAP null mice after permanent MCAO, further confirmed that astrocytes act in a neuroprotective manner in the postischemic brain (Nawashiro et al. 2000)

In the present study, we investigated whether GAS inhibits Zn^2+^-induced oxidative stress in astrocytes in the postischemic brain using a rat model and sought to identify the molecular mechanisms responsible for its protective effects, particularly with respect to Nrf2 activation and the suppressions of NADPH oxidase, PARP-1 induction, and PAR formation in C6 astroglial cells.

## Materials and methods

### Experimental animals

Male Sprague–Dawley (SD) (8–9 weeks) rats were housed under controlled diurnal lighting conditions with free access to food and tap water. All animal studies were carried out in strict accordance with the Guide for the Care and Use of Laboratory Animals published by the National Institute of Health (NIH, USA 2011) and with ARRIVE guidelines (http://www.nc3rs.org/ARRIVE). The protocol of the animal experiment was reviewed and approved by the INHA University-Institutional Animal Care and Use Committee (INHA-IACUC) with respect to ethicality (Approval Number INHA-141124-337). MCAO was carried out as previously described (Kim et al. 2011).

### Drug injection

GAS was purchased from Sigma-Aldrich (Sigma, St. Louis, MO), dissolved in dimethyl sulfoxide (DMSO) and administered intraperitoneally at 1 or 6 h post- MCAO. Rats were randomly allocated to five groups: a MCAO group, treatment-naive MCAO controls (*n* = 13); a MCAO + GAS group, GAS-treated MCAO group (*n* = 53), a GAS group, GAS-treated controls (*n* = 4), a sham group, animals underwent surgery but not MCAO (*n* = 4); and a normal group, treatment-naive control group (*n* = 4). No animals died during surgery, but the mortality rate after surgery was 4.9% (4/82).

### Infarct volume assessment

To measure infarct volumes, rats were sacrificed at 24 h post-MCAO and 2 mm brain coronal sections were obtained using a metallic brain matrix (RBM-40000, ASI, Springville, UT). Brain sections were immediately immersed in 2% 2,3,5-triphenyltetrazolium chloride (TTC; Sigma, St. Louis, MO) for 15 min at 37°C and stored in 4% paraformaldehyde (PFA; Sigma, St. Louis, MO). Infarct volumes were measured using the Scion Image Program (Frederick, MD). To account for edema and shrinkage, volumes of ischemic lesions were calculated as (contralateral hemisphere volume – (ipsilateral hemisphere volume – measured injury volume)). Infarct volumes were quantified (in mm^3^) by multiplying summed infarct areas of consecutive sections by section thickness.

### Behavioral testing

Neurological deterioration was evaluated using modified Neurological Severity Scores (mNSSs) at 24 h post-MCAO, as previously described (Kim et al. 2011). mNSS were calculated by summing the motor, sensory, reflex and balance test scores, and ranges from 0 to 18 (0 = normal, 18 = maximum deficit).

### C6 astroglial cell culture and Zn^2+^ treatment

C6 astroglial cells were grown in Dulbecco’s modified Eagle’s medium (DMEM; Sigma, St. Louis, MO) supplemented with 5% fetal bovine serum (FBS; Thermo, Waltham, MA) at 37°C in a humidified 5% CO_2_/95% air and atmosphere. C6 cells (5 × 10^4^/well) were treated with 10, 25, 50 or 100 μM of zinc sulfate (Sigma, St. Louis, MO) for 6, 12, or 24 h in serum-free DMEM or with 25, 50, 100, or 200 μM of Zn^2+^ for 15 min in HCSS (HEPES controlled salt solution).

### Reactive oxygen species quantifications

C6 cells (5 × 10^4^) were seeded into 24-well plates, cultured for 24 h, treated with Zn^2+^ and/or GAS for the indicated times, and incubated in DMEM containing 5 μM of 5-(and-6)-chloromethyl-2’,7’-dichlorodihydrofluorescein diacetate (CM-H2DCFDA; Thermo Fisher Scientific, Waltham, MA) for 30 min. After washing in PBS, fluorescence and differential interference contrast images were obtained using a Zeiss microscope (Oberkochen, Germany). Fluorescence intensity was quantified using ImageJ software (http://rsbweb.nih.gov/ij/).

### Cell viability assays

After Zn^2+^-treatments, cell viabilities were determined using a MTT (3-[4,5-dimethylthiazol-3-yl] 2,5-diphenyltetrazolium bromide; Sigma, St. Louis, MO) assay. After Zn^2+^-treatment for 24 h, C6 cells were incubated with 500 μg/ml of MTT for 60 min. Formazan crystals were solubilized using 300 μl of DMSO (Sigma, St. Louis, MO) and optical densities were read at 550 nm.

### Immunoblot analysis

C6 cells were washed with cold PBS and lysed with RIPA buffer (50 mM Tris–HCl (pH 7.4), 0.5% NP40, 0.5% Triton X-100, 0.25% sodium deoxycholate, 150 mM NaCl, and 1 complete Mini protease inhibitor cocktail tablet (Roche Diagnostics, Basel, Switzerland)). Cell lysates were centrifuged at 17,500 g for 10 min at 4°C. Total protein contents were measured using BCA protein assay kits (Thermo Fisher Scientific, Waltham, MA). The primary antibodies used were as follows: anti-PARP-1 (1: 3000; Santa Cruz Biotechnology, Santa Cruz, CA), anti-p67 (1:3000; Santa Cruz Biotechnology, Santa Cruz, CA), anti–PAR (1:3000; Trevigen, Gaithersburg, MD) and anti-α-Tubulin (1:5000; Cell Signaling, Danvers, MA). Primary antibodies were detected using a chemiluminescence kit (Merck Millipore, Darmstadt, Germany) using horseradish peroxidase-conjugated secondary antibody (1:4000; Merck Millipore, Darmstadt, Germany).

### Measurement of NAD levels

C6 cells were treated with 0.125 ml of 0.5 N HClO_4_ (Sigma, St. Louis, MO), which was then neutralized with KOH (125 mM). Supernatants obtained by centrifuging for 5 min at 10,000 g were reacted with a premix containing 0.9 mM phenazine ethosulfate (Sigma, St. Louis, MO), 0.1 mM MTT, 5.8% ethanol, 0.5 mM EDTA, and 4 U of alcohol dehydrogenase (Sigma-Aldrich, St. Louis, MO) in 61 mM Gly–Gly buffer (pH 7.4), in the dark at 37°C for 30 min. Absorbance was measured at 550 nm and result was normalized with respect to protein concentrations determined using a BCA assay kit (Thermo Scientific, Waltham, MA).

### Statistical analysis

Statistical analysis was performed using by analysis of variance (ANOVA) followed by the Newman–Keuls test. Results are presented as means ± SEMs, and statistical significance was accepted at the 5% level.

## Results

### Neuroprotective effects of GAS in the postischemic brain

**Figure 1.**
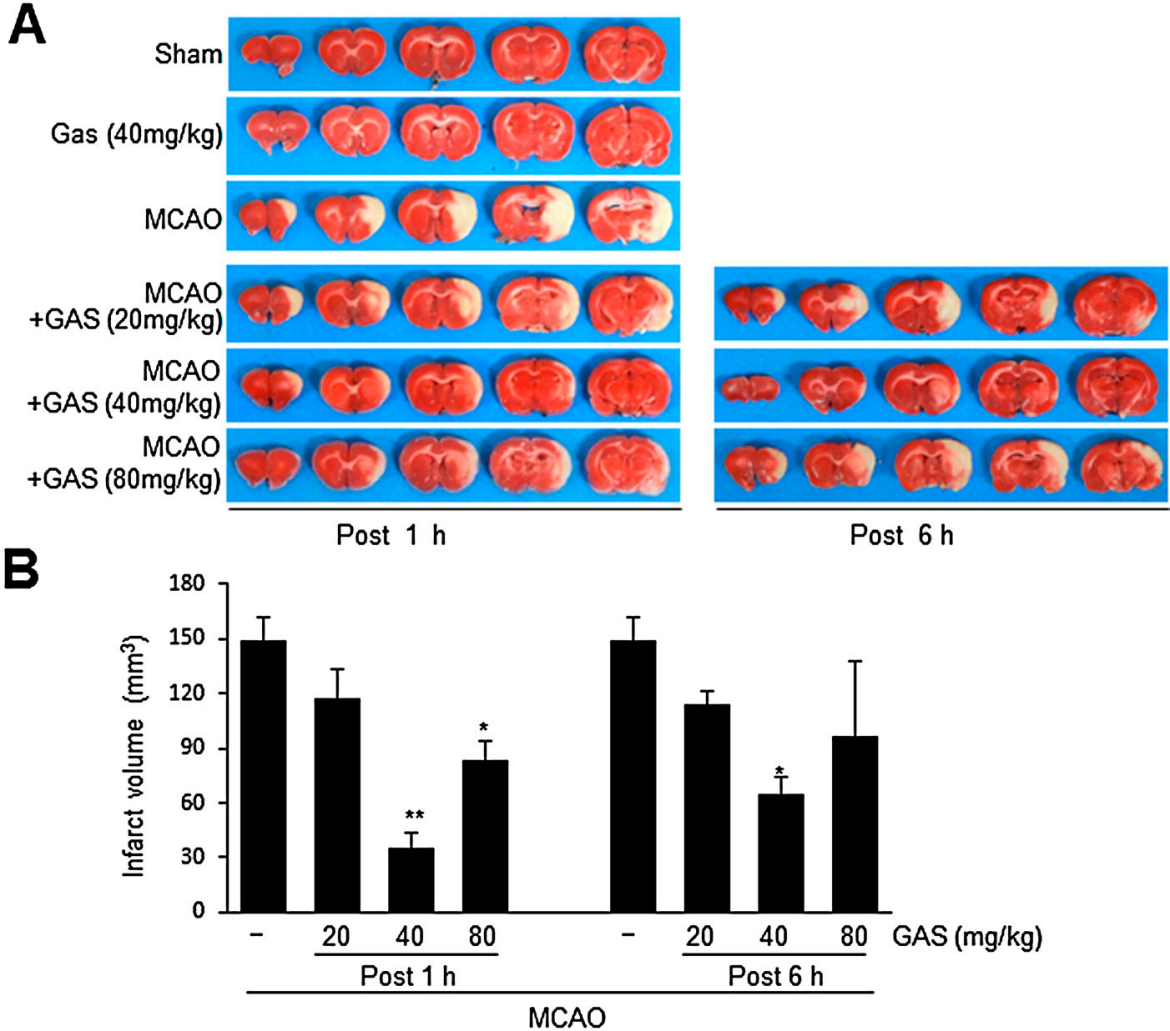
Suppression of infarct formation by GAS in the postischemic brain. GAS (20, 40, or 80 mg/kg) was administered intraperitoneally at 1 h or 6 h post-MCAO (60 min), and infarct volumes were measured by TTC staining. (B) Mean infarction volumes were measured at 1 d post-MCAO. Representative images of infarction in coronal brain sections are presented (A) and quantitative data are presented as means ± SEMs (B). Sham, sham-operated control rats (*n* = 4); GAS, GAS-administered control rats (*n* = 4); MCAO, treatment-naïve MCAO control rats (*n* = 8); MCAO + GAS, GAS-administered MCAO rats (*n* = 29). *p < 0.05, **p < 0.01 versus treatment-naïve MCAO controls.

GAS (20, 40, or 80 mg/kg) was administered intraperitoneally at 1 or 6 h post-MCAO to rats and infarct volumes were assessed at 1 d post-MCAO. GAS (40 or 80 mg/kg) administered at 1 h post-MCAO reduced infarct volumes to 22.2 ± 5.6% (*n* = 5, *p* < 0.01), and 56.8 ± 12.6% (*n* = 5, *p* < 0.01), respectively, of that of treatment naïve MCAO controls (Figure 1(a and b)). When 40 mg/kg of GAS was administered at 6 h post-MCAO, mean infarct volume reduced to 43.9 ± 9.6% (*n* = 4, *p* < 0.05) of that of treatment naïve MCAO controls (Figure. 1(a and b)). These results show GAS exerted a neuroprotective effect against ischemic injury with a wide therapeutic window.

### Improvements in neurological deficits after MCAO by GAS

**Figure 2.**
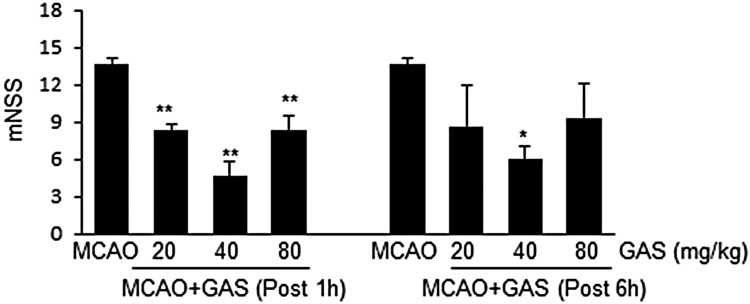
Improvement of neurological deficits by GAS. GAS (20, 40, or 80 mg/kg) was administered intraperitoneally at 1 h or 6 h post-MCAO and neurological deficits were evaluated using mNSS at 1 d post MCAO. MCAO, treatment-naïve MCAO control rats (*n* = 8); MCAO + GAS, GAS-administered MCAO rats (*n* = 27). *p < 0.05, **p < 0.01 versus treatment-naïve MCAO controls.

mNSSs were measured at 1 d post MCAO. The mean mNSS score of treatment-naïve MCAO controls was 13.7 ± 0.4 (*n* = 8) (Figure 2). The administration of GAS at 20, 40, or 80 mg/kg at 1 h post-MCAO reduced mNSSs to 8.3 ± 0.5 (*n* = 7), 4.3 ± 0.9 (*n* = 4), and 8.3 ± 0.9 (*n* = 4), respectively (Figure 2), and GAS at these same levels administered at 6 h post-MCAO reduced mNSS scores to 9.0 ± 2.3 (*n* = 4), 6.0 ± 0.7 (*n* = 4), and 8.5 ± 2.1 (*n* = 4), respectively (Figure 2). Accordingly, GAS administration was found to be accompanied by marked reductions in neurological deficits. In addition, we found pH, PaO_2_, PaCO_2_, and glucose levels were similar in GAS-treated and -untreated animals (Table 1).

**Table 1.** Physiological parameters.

	Control group		Ischemia group	
	Base	GAS	MCAO	MCAO + GAS
Temperature, °C	37.08 ± 0.17	37.0 ± 0.13	37.04 ± 0.14	37.04 ± 0.12
pH	7.64 ± 0.04	7.42 ± 0.00	7.58 ± 0.03	7.40 ± 0.02
pO_2_,mmHg	145.8 ± 19.2	163.2 ± 12.6	150.22 ± 6.66	166.6 ± 9.77
pCO_2_,mmHg	32.16 ± 1.95	32.3 ± 0.96	39.06 ± 1.23	30.04 ± 0.70
Glucose, mg/dL	109.80 ± 5.63	103.4 ± 2.92	109.6 ± 5.19	95.6 ± 6.90

Values are mean SD (*n* = 4). One-way analysis of variance revealed no significant intergroup difference for any variable.

### Suppression of Zn^2+^-induced C6 astroglial death by GAS

**Figure 3.**
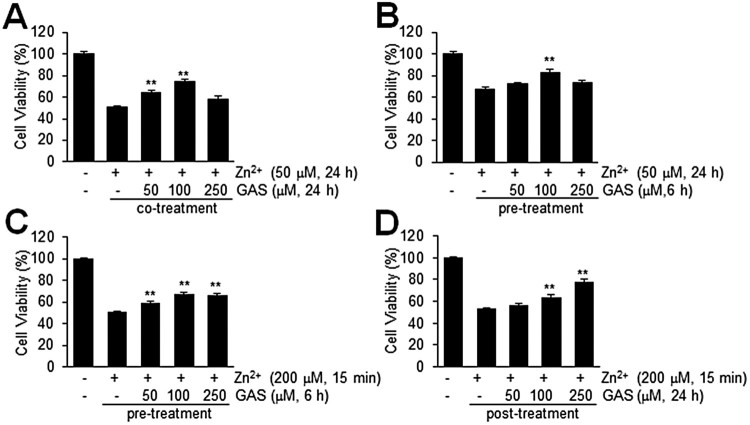
Increased cell viability by GAS in Zn^2+^-treated C6 cells. (A, B) C6 cells were co-treated with 50 μM of Zn^2+^ and GAS (50, 100, or 250 μM) for 24 h (A) or treated with 50 μM of Zn^2+^ for 24 h with or without pre-treatment of GAS (50, 100, or 250 μM) for 6 h (B). (C, D) C6 cells were treated with 200 μM of Zn^2+^ for 15 min with or without 6 h of GAS pretreatment (50, 100, or 250 μM) (C) or post-treated with GAS at these concentrations for 24 h (D). Cell viabilities were measured using a MTT assay. Results are presented as means ± SEMs (*n* = 4) **p < 0.01 versus Zn^2+^-treated controls.

To determine whether GAS has protective effects protects against Zn^2+^-toxicity, we examined the viabilities of C6 cells, an astrocyte cell line, after treating them with Zn^2+^ for 15 min to 24 h in the presence or absence of GAS. When C6 cells were treated with Zn^2+^ (50 μM) for 24 h, mean cell viability was 50.9 ± 2.8% (*n* = 4) (Figure 3(a)). However, co-treatment with GAS (50, 100, or 250 μM) significantly increased cell viability, and at a GAS concentration of 100 μM, mean cell viability after 24 h was 74.9 ± 3.7% (*n* = 4) (Figure 3(a)). In addition, pre-treating C6 cells with GAS (50, 100, or 250 μM) for 6 h before Zn^2+^ treatment increased cell viability slightly (Figure 3(b)). Similarly, C6 cell viability after acute Zn^2+^ treatment (200 μM, 15 min) was also enhanced to 67.7 ± 5.6% (*n* = 4) by pre-treating cells with GAS (100 μM, 6 h) or to 77.8 ± 7.0% (*n* = 4) by post-treating cells with GAS (250 μM, 24 h), respectively, as compared with that of treatment-naïve Zn^2+^ control cells, 50.8 ± 2.0% (*n* = 4) or 53.3 ± 3.3% (*n* = 4) (Figure 3(c and d)). These results shows GAS had a prominent protective effect against acute or chronic Zn^2+^-induced C6 cell death.

### Up-regulation and nuclear translocation of Nrf2 by GAS in C6 astroglial cells

**Figure 4.**
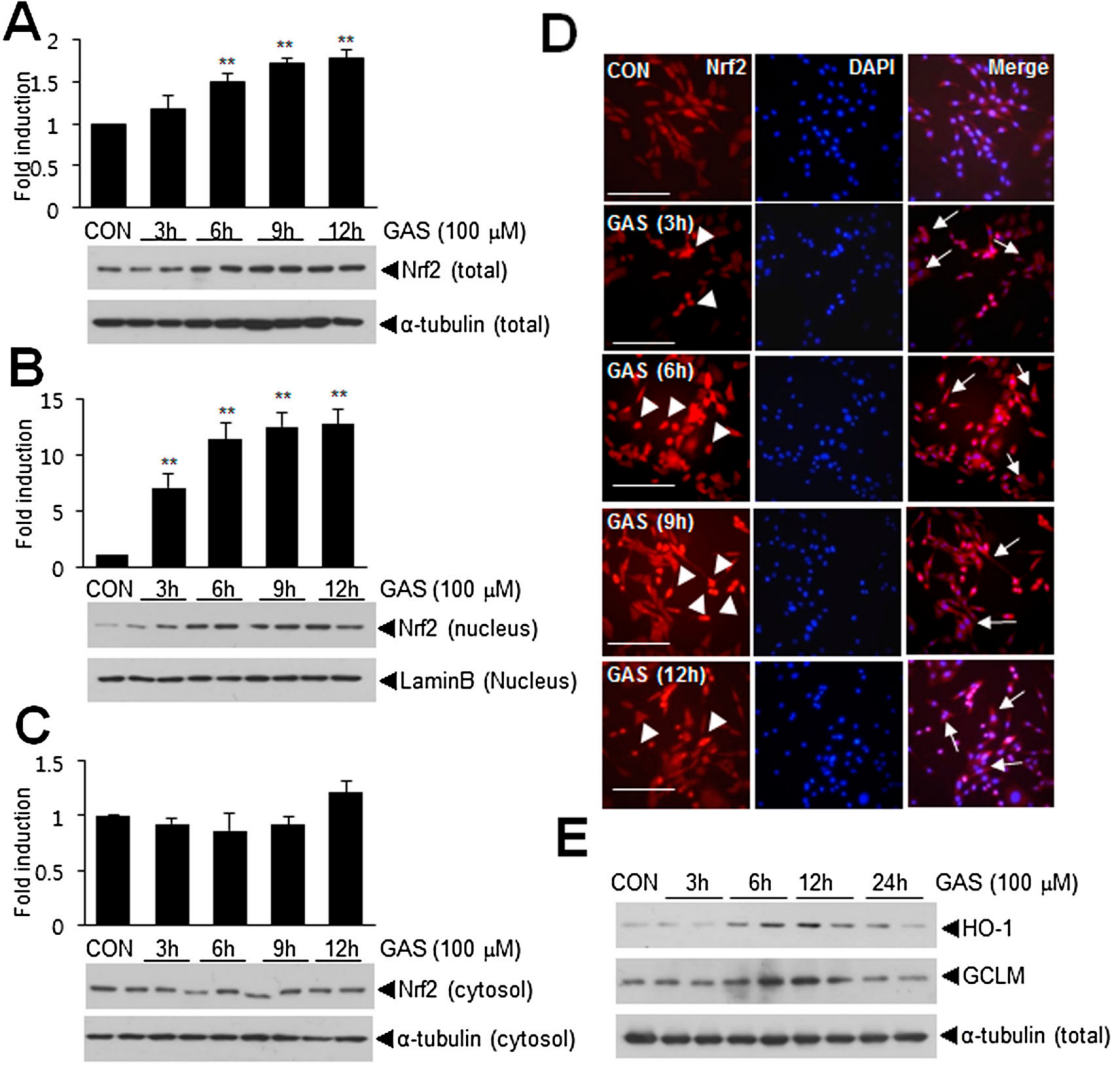
The up-regulation and nuclear translocation of Nrf2 by GAS. (A, B) C6 cells were treated with 100 μM of GAS for 3, 6, 9, or 12 h and total (A), nuclear (B) and cytoplasmic Nrf2 levels (C) were determined by immunoblotting. (D) Double fluorescent staining was performed using anti-Nrf2 antibody and DAPI after treating C6 cells with GAS (100 uM) for 3, 6, 9, or 12 h. Arrowheads indicate Nrf2 translocation from cytoplasm to nucleus and arrows indicate cytoplasmic localization of Nrf2. (D) C6 cells were treated with 100 μM GAS for 3, 6, 9, or 12 h and protein levels of HO-1, GCLM, and α-tubulin were determined by immunoblotting. The scale bar represents 100 μm. Results are presented as means ± SEMs (*n* = 4) **p < 0.01 versus treatment naïve controls.

Since it has been reported that many phytochemicals regulate Nrf2 (Surh et al. 2005), we investigated whether GAS induces the expression and/or the nuclear translocation of Nrf2 in C6 cells. Total intracellular levels of Nrf2 started to increase after 3 h of GAS (100 μM) treatment and then progressively increased over the following 3, 6, 9, or 12 h (Figure 4(a)). Furthermore, the nuclear translocation of Nrf2 was detected after 3 h of GAS treatment, and its nuclear levels further increased of the next 9 h (Figure 4(b)). Double fluorescence immunostaining with anti-Nrf2 and DAPI showed that GAS induced the nuclear translocation of Nrf2 at 3 h and further increased the nuclear translocation for 9 h, when Nrf2 was also detected in cytoplasm (Figure 4(d)). Expressions of anti-oxidative genes, e.g. HO-1 and GCLM, were also induced by GAS (100 μM) (Figure 4(e)). These results indicated that GAS induced the up-regulation and nuclear translocation of Nrf2 and increased the expression of downstream anti-oxidative genes.

### GAS inhibited ROS generation and NAD depletion in Zn^2+^-treated C6 cells

**Figure 5.**
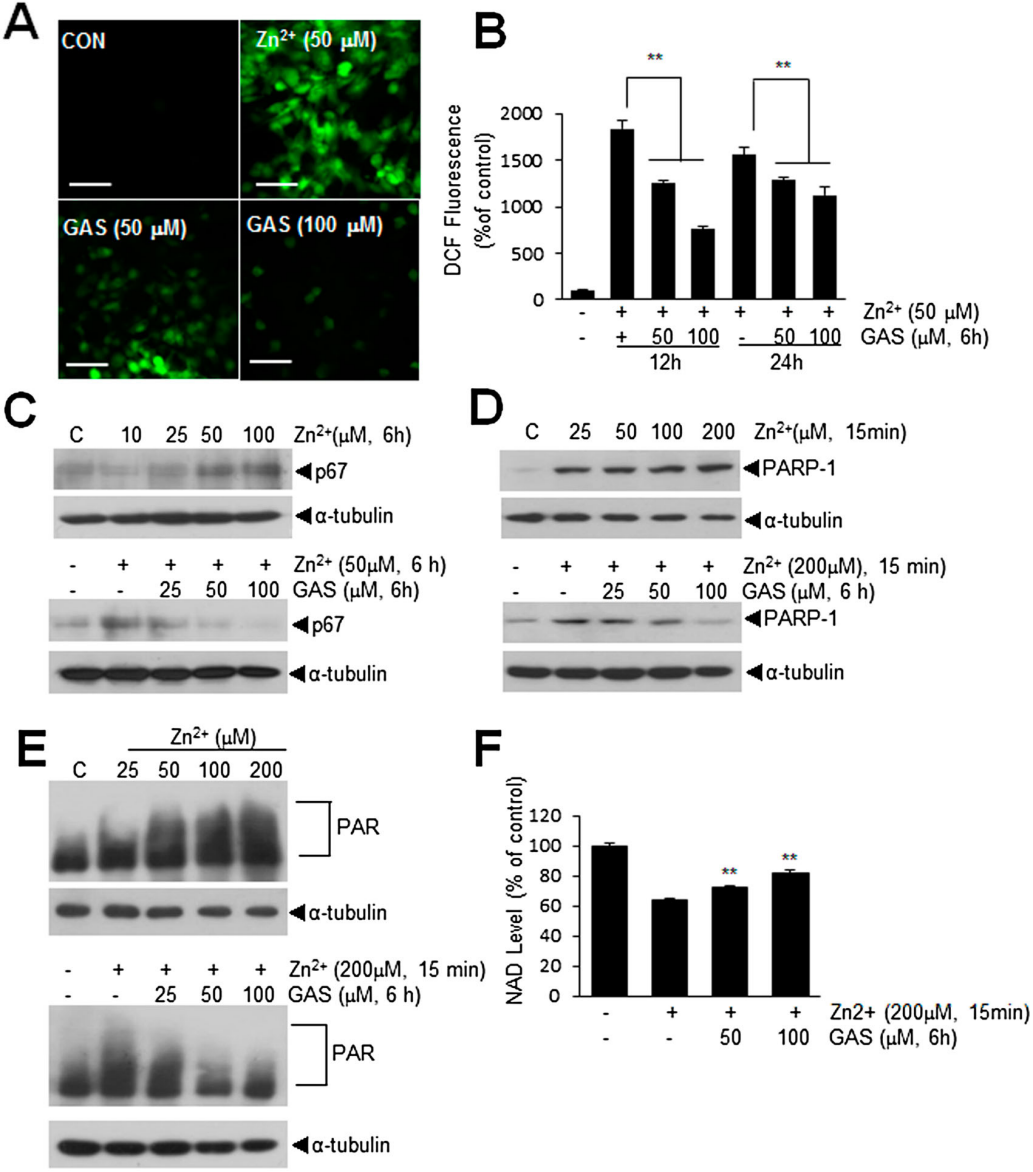
Suppressions of Zn^2+^-mediated inductions of ROS, p67, and PARP-1in C6 astroglial cells. (A, B) C6 cells were treated with Zn^2+^ (50 μM) for 12 or 24 h with or without pre-treating GAS (50 or 100 μM) for 6 h, and intracellular ROS productions were measured using CM-H2DCFDA. (C) Protein levels of p67 were measured after 6 h of Zn^2+^ treatment (10, 25, 50, 100 μM) and after 50 μM of Zn^2+^ treatment with pre-treating cells with GAS (25, 50, 100 μM) for 6 h. (D-E) PARP-1 and PAR levels were measured at 60 min after Zn^2+^ (25, 50, 100, 200 μM) treatment and at 60 min after 200 μM of Zn^2+^ treatment with pre-treating GAS (25, 50, 100 μM) for 6 h prior to Zn^2+^ treatment. (F) NAD levels were measured in C6 cells at 6 h after Zn^2+^-treatment (200 μM, 15 min) with or without GAS pre-treatment (50 or 100 μM, 6 h). Quantitative data are presented as means ± SEMs. **p < 0.01 versus Zn^2+^-treated controls. The scale bar represents 50 μm.

Since, it has been reported excessive Zn^2+^ triggers ROS production (Noh and Koh 2000), we examined Zn^2+^-induced ROS production in C6 cells in the presence or absence of GAS. Intracellular ROS levels were visualized using CM-H2DCFDA and found to be significantly increased after 12 h of Zn^2+^ (50 μM) treatment (Figure 5(a)). However, pre-treatment with 50 or 100 μM of GAS for 12 h reduced Zn^2+^- induced ROS production to 68.8 ± 4.7% and 41.6 ± 3.7%, respectively (Figure 5(b)). Similarly, it was reduced to 83.14 ± 5.7% and 72.8 ± 9.0%, respectively, measured at 24 h after Zn^2+^ (50 μM) treatment (Figure 5(b)). Zn^2+^ (50 μM) was also observed to increase p67 (an NADPH oxidase subunit) levels in C6 cells (Noh and Koh 2000) and these were also suppressed by GAS (100 μM) pre-treatment (Figure 5(c)). In addition, it has been reported Zn^2+^ also induces PARP-1 activation, which depletes NAD+ and ATP and causes cell death (Sheline et al. 2003). PARP-1 up-regulation was observed in Zn^2+^-treated C6 cells and found to be significantly suppressed by pre-treating cells with GAS (50 or 100 μM) for 6 h (Figure 5(d and e)). Furthermore, subsequent PAR formation and NAD depletion by Zn^2+^ were suppressed by pre-treating cells with GAS at 50 or 100 μM for 6 h. These results indicate GAS suppressed Zn^2+^-induced ROS production and PAR formation by inhibiting p67 and PARP-1 induction in Zn^2+^-treated C6 cells.

## Discussion

GAS is a phenolic glycoside and a main bioactive constituent of Rhizoma Gastrodiae, which has been traditionally used to treat various diseases. Furthermore, numerous scientific studies have reported GAS has neuroprotective effects in CNS disorders, such as, Alzheimer’s disease (Hu et al. 2014), Parkinson’s disease (Wang et al. 2014), and cerebral ischemia (Zeng et al. 2006). In particular, GAS has been reported to have neuroprotective effects in an animal model of transient MCAO, wherein GAS (15, 30, and 60 mg/kg) was pre-treated for 7 d (Liu et al. 2016). However, in the present study, we observed a greater neuroprotective effect after a single bolus (40 mg/kg, i.p.) administration of GAS at 1 or 6 h after MCAO (Figure 1(b)). In addition, we found GAS protected astrocytes from Zn^2+^ toxicity and had anti-oxidative effects. Although GAS has been previously reported to exhibit prominent neuroprotective effects in a MCAO model (Liu et al. 2016), we are the first to report that it protects astrocytes against toxic effects of Zn^2+^, which might contribute to its robust protective effects in MCAO animal model.

During the early stage after stroke, massive neuronal cell damage is caused by excitotoxicity and Zn^2+^ overload, (Lipton 1999) and subsequent inflammation and apoptosis over a few hours to days aggravate ischemic brain damage (Graham and Chen 2001). In neurons, excessive Zn^2+^ accumulation and its oxidative and excitotoxic effects cause severe damage by inhibiting GAPDH, which reduces glycolysis and NAD levels (Sheline et al. 2000). Although astrocytes are more resistant than neurons to high levels of Zn^2+^ (Dineley et al. 2000), ROS production and glutathione formation are nevertheless impaired (Noh and Koh 2000; Bishop et al. 2007). In the present study, we found GAS markedly suppressed Zn^2+^-induced ROS production and Zn^2+^-induced elevation of the levels of the p67 subunit of NADPH oxidase in astrocytes, indicating the anti-oxidative effects of GAS underlie, at least in part, its amelioration of the toxic effects of Zn^2+^ in astrocytes.

The anti-oxidative effects of GAS have been reported in several cell types including neurons, in which Nrf2 (Wang et al. 2014), HO-1, p38 MAPK (Jiang et al. 2014), and ERK (Zhao et al. 2012) were shown to be involved. In the present study, GAS increased Nrf2 expression and its nuclear translocation in C6 astrocytes and maintained this over 12 h of treatment (Figure 4(a and b)) and also induced the expressions of antioxidant genes, such as, HO-1 and GCLM (Figure 4(c)). However, we found astrocytes need to be treated with GAS at a higher dose (100 μM) compared to other cell types in previous studies (25–30 μM) (Zhao et al. 2012) and it might be due to cell type differences. In addition, rapid nuclear translocation of Nrf2 induced by GAS prompted us to speculate GAS-mediated Nrf2 translocation might be able to inhibit inflammatory response by promoting p300 to Nrf2 binding rather than p300 to p65 binding, as we previously reported for ethyl pyruvate and curcumin (Kim et al. 2013; Lee et al. 2016). Further study is needed to confirm that the anti-inflammatory effects exert by this mechanism also contribute to the neuroprotective effect of GAS.

We also observed GAS significantly suppressed Zn^2+^-mediated PARP-1 induction and subsequent PAR formation in astrocytes (Figure 5(e)). Although, astrocytes are more resistant to ischemia than neurons, excess Zn^2+^ release from a subset of neurons after stroke promotes astrocyte hypoxic cell death by modifying PARP-1 and upregulating HIF-1α (Pan et al. 2013). Furthermore, PARP-1 activation inhibits glutamate uptake by astrocytes and leads to NAD+ and ATP depletion (Suh et al. 2007). We also showed increase in NAD levels in astrocytes by GAS. Therefore, astrocyte damage induced by excessive Zn^2+^ release from neuron further exacerbates neuronal damage through this vicious cycle in the postischemic brain. Since astrocytes are known to have beneficial effects in the postischemic brain, such as, anti-excitotoxic effects, and releasing neurotrophins during the acute phase, and contribute to angiogenesis, neurogenesis, and synaptogenesis during the recovery phase (Liu et al. 2016), GAS-mediated protective effects on astrocytes contribute to its robust neuroprotective effects observed in MCAO model.

Taken together, our results show GAS suppressed Zn^2+^-induced astrocytic cell death by inhibiting the inductions of p67 and PARP-1, and that *in vivo* suppressed infarct formation, and improved neurological deficits. Because Zn^2+^ toxicity is a known component of various neurological diseases, such as, trauma and epilepsy, we suggest further studies be undertaken to investigate the merits of GAS in targeting Zn-induced toxicity.
